# Editorial: Probing the Chromatin Architecture

**DOI:** 10.3389/fcell.2021.727803

**Published:** 2021-07-29

**Authors:** Francesca Palombo, Stefano Pagliara, Ankur Singh, Richard Chahwan

**Affiliations:** ^1^School of Physics and Astronomy, University of Exeter, Exeter, United Kingdom; ^2^Living Systems Institute and School of Biosciences, University of Exeter, Exeter, United Kingdom; ^3^Woodruff School of Mechanical Engineering, Georgia Institute of Technology, Atlanta, GA, United States; ^4^Coulter Department of Biomedical Engineering, Georgia Institute of Technology and Emory University School of Medicine, Atlanta, GA, United States; ^5^Institute of Experimental Immunology, University of Zurich, Zurich, Switzerland

**Keywords:** chromatin organization, cell phenotyping, genome, cancer therapy, vibrational spectroscopy, imaging, confocal microscopy

Chromatin architecture plays an essential role in gene regulation, cell differentiation, response to external cues, and disease progression, and hence can be used as a biomarker for cell phenotyping. Traditionally, methods based on confocal microscopy and imaging using fluorescent tags have been employed to study the chromatin architecture inside the nucleus by measuring the location and folding of chromosomes. However, existing methods lack specificity and/or are reliant on extrinsic labeling that prevents yielding structural information for chromatin in its native state by potentially interfering with ongoing biological processes. In this Research Topic, we focus on technical advancements in (i) measuring chromatin changes at the single-cell level, (ii) studying chromatin modification through DNA methylation, and (iii) visualizing chromatin by super-resolution microscopy. We also review the literature on (i) protein post-translational modifications and their impact on the scaffold/matrix interactions of the chromatin, (ii) advancements in using epigenomic biosensors during radiotherapy, and (iii) the effect of small molecule inhibitors on chromatin-associated cellular responses.

Morrish et al. describe a label-free imaging approach based on two complementary chromatin probing methods—Raman microscopy in microfluidic devices and transcriptomic analysis—that enables direct linking of Raman spectral signatures of the nucleus of lymphocyte cells with >17,000 distinct transcripts upon immune activation of B lymphocytes. This approach, built on a previous method based on Raman spectroscopy, was applied to image >100 activated and non-activated live B cells at high resolution in a specially designed microfluidic chip. This complex set of cellular Raman maps was analyzed via a multivariate common k-means cluster analysis approach. This analysis permitted identifying distinct spectral profiles associated with the nucleus and cytoplasm. Next, principal component analysis (PCA) and linear discriminant analysis (LDA) allowed elucidating the discriminants between activated and non-activated cells. This label-free imaging classification has exciting potential in biology. For instance, it can be used in cell sorting based on chemical phenotyping of the nucleus, or in clinical translation for label-free disease diagnosis and prognosis. Finally, the team quantified linear correlations between nuclear Raman spectra and transcriptomic data, generating a partial least squares (PLS) regression matrix to predict Raman data from transcriptomic profiles. This approach was used to identify the key transcripts and thereby reveal genes or pathways that are essential for the immune activation process, including the immunoglobulin genes *Ighm* and *Igha*, regulatory RNAs and proteins.

An alternative approach for tracing chromatin dynamic changes used genome-wide quantification of DNA methylation in induced pluripotent stem cells (iPSCs) during differentiation and maturation into cortical neurons. This method, based on data-driven trajectory inference, allows identification of DNA methylomic trajectories of neuronal differentiation. Up to 6,843 Bonferroni-significant loci (out of the 41,851 loci measured) were identified based on the presence of progressive alterations in DNA methylation during differentiation into neurons and subsequent maturation. By applying a gene-gene interaction network analysis, the authors were able to identify 60 densely connected genes that were influential in neuronal differentiation from iPSCs, particularly those encoding transcription factors and epigenetic regulators (Imm et al.).

A complementary approach for visualizing chromatin architecture and its epigenomic states with nanometric resolution in cells and tissue used super-resolution imaging based on single molecule localization microscopy (SMLM), as reported in this review (Xu and Liu). SMLM-based super-resolution imaging of nanoscale chromatin architecture and its dynamic behaviors was applied to study healthy and pathological processes in developmental biology, immunology, and oncology. The authors propose that in the future the SMLM imaging approach for tracing chromatin architecture could improve cancer diagnosis, enabling the development and evaluation of new early detection strategies.

Chromatin architecture is based on the interaction between DNA and histones as well as the post-translational modifications (PTMs) of non-histone-associated proteins. Among the main proteins responsible for maintaining the 3D genomic architecture, S/MAR-binding proteins (S/MARBPs) play a key role in binding to the scaffold/matrix attachment regions (S/MARs) and mediating tethering of the chromatin to the protein matrix. A review of the current understanding, scope, disease implications, and future perspectives of the diverse post-translational modifications that regulate the functions of S/MARBPs is featured here (Roychowdhury and Chattopadhyay).

Finally, approaches relying on a combination of drugs affecting the epigenomic landscape and radiotherapy along with chromatin epigenetic monitoring based on fluorescence resonance energy transfer (FRET) biosensors may lead to improvement in cancer treatment. However, although beneficial, such approaches have been evaluated in several ongoing clinical trials for limited cancer types, partly due to a lack of knowledge on the mechanisms on radiation-induced epigenomic regulation and chromatin remodeling. Peng et al. review recent advances in radiotherapy and epigenomic remodeling and propose that, in the future, a FRET-based approach could be used for epigenetic monitoring and chromatin architecture probing in tumor cells upon exposure to radiation, and for screening drugs that influence the epigenomic landscape which, when combined with radiotherapy, can improve cancer treatment.

One example of such epigenetic drugs, valproic acid (VPA), was originally prescribed for the treatment of seizure disorders and is now known to affect epigenetic markers and chromatin structure, as reviewed here (Mello). VPA indirectly promotes histone acetylation by inhibiting the deacetylation process, and therefore it can induce chromatin decondensation, as revealed through imaging techniques such as Fourier Transform Infrared (FTIR) spectroscopic imaging. This finding suggests that VPA could affect the methylation status of histones, and possibly DNA, and hence could have therapeutic potential due to its antitumour effects.

## Author Contributions

FP wrote the manuscript with support from SP, AS, and RC. All authors contributed to the article and approved the submitted version.

## Conflict of Interest

The authors declare that the research was conducted in the absence of any commercial or financial relationships that could be construed as a potential conflict of interest.

## Publisher's Note

All claims expressed in this article are solely those of the authors and do not necessarily represent those of their affiliated organizations, or those of the publisher, the editors and the reviewers. Any product that may be evaluated in this article, or claim that may be made by its manufacturer, is not guaranteed or endorsed by the publisher.

